# The inhibition by human MSCs-derived miRNA-124a overexpression exosomes in the proliferation and migration of rheumatoid arthritis-related fibroblast-like synoviocyte cell

**DOI:** 10.1186/s12891-020-3159-y

**Published:** 2020-03-06

**Authors:** Hong-Yan Meng, Li-Qing Chen, Li-Hui Chen

**Affiliations:** 1 Department of Rheumatology and Immunology, Shandong Provincial Hospital Affiliated to Shandong University, Jinan, Shandong 250021 P.R. China; 2 Department of Traditional Chinese Medicine, Shandong Provincial Hospital Affiliated to Shandong University, Jinan, Shandong 250021 P.R. China; 3 Health Management Center of Shandong Sunshine Union Hospital Co.,Ltd., Shandong, P.R. China

**Keywords:** Rheumatoid arthritis, Exosome, miRNA-124a, Mesenchymal stem cells, Fibroblast-like Synoviocytes

## Abstract

**Background:**

Rheumatoid arthritis is a long-term, progressive autoimmune disease. It is characterized by synovial hyperplasia leading to swelling, stiffness, and joint deformity in more than one joint. Fibroblast-like synoviocytes are the major cell types that make up the synovial intima structure, which is one of the decisive factors in the development and course of rheumatoid arthritis.

**Methods:**

The potential therapeutic effects of MSCs-derived miRNA-124a overexpression exosomes were evaluated in vitro by the method including MTT assay and cell cycle test for cell proliferation, scratch wound closure and transwell for cell migration, flow cytometry and western for the apoptosis detection.

**Results:**

Exosomes derived from human MSCs that overexpression miRNA-124a were prepared and characterized. We found that the pretreatment of this exosome was able to inhibit the proliferation and migration of fibroblast-like synoviocyte cell line and promote the apoptosis of this cell during the co-incubation.

**Conclusions:**

Exosomes derived from MSCs were proved to be a suitable vector for the delivery of therapeutic miRNA-124a, and such miRNA-124a overexpression exosomes were expected to provide a new medicine and strategy for the treatment of rheumatoid arthritis.

## Background

Rheumatoid arthritis (RA) has a high rate of joint deformities and disability, which puts a heavy burden on medical services around the world [1]. Synovial hyperplasia is a pathological feature of rheumatoid arthritis, which leading to progressive cartilage and bone destruction [2]. Fibroblast-like synoviocytes (FLS) are the major cell types that make up the synovial intima structure, which is of great importance in the pathogenesis of RA [3]. Activated FLS secretes large numbers of inflammatory cytokines, chemokines and metalloproteinases, which lead to proliferative inflammation of the synovium, massive angiogenesis and destruction of cartilage and bone [4]. It is now widely accepted that the activation of FLS is one of the decisive factors in the development and course of RA [5].

miRNAs regulate many physiological processes at the transcriptional and post-transcriptional level [6]. The aberrant expression of certain miRNAs is associated with several human diseases [7–9]. Increasing evidence has revealed that altered expression of miRNA in FLS and synovial tissue is closely related to the development of RA [10]. Takuya Niimoto et al. reported that miR-146a was closely related to IL-17 regulation in the FLS in RA patients [11]. Joanna Stanczyk et al. reported that the increasing levels of miR-203 was responsible for the increasing concentration of MMP-1 and IL-6 and thereby lead to the activation of synovial fibroblasts in RA [12]. All the above mentioned suggest that miRNA plays an important role in the development of RA, which may become a potential therapeutic target in the treatment of RA.

The previous study showed that miRNA-124a suppressed the cell proliferation and migration of hepatoma carcinoma cell [13]. Moreover, miR-124a is involved in the migration and invasion of glioblastoma [14]. Others reported that miRNA-124a was a key regulator of proliferation in FLS, which suggested the potential value for therapy of RA [15]. However, an effective way is required for the therapeutic miRNA to inter target cells. Zhe Chen et al. has reported that miR-150-5p–overexpressing Mesenchymal stem cells (MSCs) derived exosomes reduced joint destruction by inhibiting synoviocyte hyperplasia and angiogenesis, which presented a new strategy of stem cell-derived drugs and miRNA delivery [16]. MSCs was able to differentiate into multiple cell lines and have a strong self-renewal, immunosuppression and damage repairing ability [17]. Research from Liming Wang et al. indicated that treatment with DMARDs plus UC-MSCs (umbilical cord mesenchymal stem cells) was able to provide long-lasting clinical benefits for patients with active RA [18]. However, the immunosuppressive of MSCs infusion tend to promote tumor growth in allogeneic animals, and exert a potential risk of tumorigenesis [19]. Moreover, the survival time and biological activity of MSCs cannot ideally under control in vivo. Exosomes from MSC are important carriers for signal exchange with target cells which was able to partially mimic the function of MSC [20, 21]. Therefore, exosome form MSC not only can exert the therapeutic effects of stem cells, but also a perfect vector for the delivering of miRNA.

In this study, we combined the potential value of miRNA-124a and MSC in the treatment of rheumatoid arthritis and generated HMSC-124a-EV (miR-124a overexpressing human MSC-derived exosomes). The potential therapeutic effects of HMSC-124a-EV were evaluated in vitro with the rheumatoid arthritis-related fibroblast-like synoviocyte cell line, MH7A cells. The change in cell cycle stages, migration capabilities, and apoptosis-related gene expression were studied after the co-incubation of MH7A cell lines with HMSC-124a-EV.

## Methods

### Cell culture

The Fibroblast-like Synoviocyte cell line that we used to mimic Fibroblast-like synoviocytes from rheumatoid arthritis patients in the current study named MH7A cell. MH7A cell was purchased from GuanDao Biological engineering corporation (Shanghai, China). Human MSCs were bought from National Infrastructure of Cell Line Resource (China, 3153C0001000000244). It was isolated from the bone marrow of a 26-year-old man in 2017. Cells were individually cultured in Dulbecco’s Modified Eagle’s Medium (DMEM, Hyclone), supplemented with 10% fetal bovine serum (FBS, Gibco) and 100 U/mL penicillin/streptomycin (Gibco), at 37 °C in a humidified 5% CO_2_ incubator.

### The preparation of exosomes form human MSC

The exosomes that carry microRNA-124a was derived from human MSCs (HMSCs). Adenovirus that carries a microRNA-124a over-expression vector was used to transfecting HMSCs in this study. The adenovirus was established by Genechem (Shanghai, China), and the prepared adenovirus was added to the HMSCs medium in 6-well plate. The adenovirus that contains a blank vector was set as a negative control for all the experiments. After 24 h of co-incubation, the cells were transfected and the exosomes were prepared after another 1–2 days co-incubation. Exosomes were collected by Total Exosome Isolation Reagent (Invitrogen 4,478,359) following the manufacturer’s protocol. The exosomes were resuspended by PBS and stored at − 80 °C avoiding repeated freeze-thaw cycles. All the experiment in our study was approved by the Ethics Committee in Shandong Provincial Hospital Affiliated to Shandong University.

### qRT-PCR for miRNA-124a

The operation was performed as previous described [22]. In brief, total RNA was extracted from the MH7A cells and exosomes by Trizol (Thermo Scientific). 1 μg of RNA was reverse transcribed into cDNA by a ReverTra Ace qPCR RT Master Mix with gDNA Remover (TOYOBO) following the manufacturer’s protocol. Then, we performed amplification for 40 cycles (TOYOBO, QPS-201). The level of miRNA-124a was normalized to RNU6, and 2^−ΔΔCt^ method was used to evaluated the relative expression of gene. The primers sequence was listed as follow: U6: (5′-GCTTCGGCAGCACATATACTAAAAT-3′), miRNA-124a:(5′-TAAGGCACGCGGTGAATGCC-3′).

### MTT assay

MH7A cells were plated in 96-well plates at a density of 5 × 10^3^ cells per well.20 μg/ml HMSC-EV and HMSC-124a-EV were added and MTT Cell Proliferation and Cytotoxicity Assay Kit (Beyotime, China) was used for the cell proliferation assay at 0, 4, 8, 16, 24 and 48 h after the addition of HMSC-EV and HMSC-124a-EV, respectively.

### Flow Cytometry

For Flow Cytometry analysis, 1 × 10^5^ cells were harvested at a density of 1 × 10^6^/ml and then stained with the corresponding antibodies for 30 min at 4 °C within cell stain buffer (Biolegend, 420,201). The reaction was stopped by washing twice with cell stain buffer and then resuspend the cell with 200 μL of cell stain buffer. The positive population was gated by appropriate iso-type controls. All the antibodies used in this study were purchase from Biolegend.

### Cell cycle assay

Cell Cycle and Apoptosis Analysis Kit (Beyotime, China) was used for the cell cycle assay in this study. MH7A cells were seeded in 6-well plates at a density of 5 × 10^5^ cells per well. After overnight culture, 20 μg/ml of HMSC-EV and HMSC-124a-EV were added respectively, and continued culture for another 24 h. Cells were then harvested and fixed in 70% ethanol overnight at 4 °C. After that, the cells were stained by PI following the manufacturer’s protocol.

### Western blot

Cells were collected and washed with PBS, then lysed using the Cell lysis buffer for Western and IP (Beyotime Inc., China). The concentrations of protein extracts were analyzed with the Enhanced BCA protein assay kit (Thermo Scientific). Proteins were separated by 10% SDS-PAGE gel and then transferred onto PVDF (Merck millipore) membranes. Membranes was blocked by 5% non-fat milk and incubated with primary antibody 4°Covernight. After extensively washing, the membrane was incubated with corresponding secondary antibody (Proteintech) for 1 h. Finally, the protein bands were detected by the Enhanced Chemiluminescence (Abclone) system.

### Scratch wound closure assay

MH7A cells were seeded within 6-well plates at a density of 5 × 10^5^ cells per well. After the cells were grown to confluence, and discard the medium and wash 3 times with PBS. Then, scratch a straight line on the culture well surface with a 100-lL pipette tip and wash 3 times with PBS again. After washing, add fresh medium and 20 μg/ml of HMSC-EV or HMSC-124a-EV respectively. AxioCam single-channel camera and AxioVision software (Carl Zeiss, AG) was used to record the image every 30 min over the 24-h incubation period. The images of representative time point were analyzed by using ImageJ software, and the wound areas were measured to determine the percentage of wound closure. The percentage of wound healing can be obtained by the ratio of the wound healed area (the initial wound area minus the area at a certain time point) to the initial wound area. Randomly sampled single cell migratory pathways were also analyzed by using the “Tracking” package in AxioVision software, which was capable of the measurement for the wound healing (migration) rate.

### Migration and invasion assays

For assessment of cell migration, MH7A cells were plated at a density of 1 × 10^5^ cells per chamber in serum-free DMEM in the upper chamber of transwell plates fitted with 8-mm pore membranes. DMEM supplemented with 10% FBS and 20 μg/ml of HMSC-EV or HMSC-124a-EV was added to the lower chamber. After 24 h, nonmigrating cells were removed from the upper surface by 3 times washing with PBS and filters were stained with crystal violet. We counted the migrated cells in five different microscopic fields at 100× magnification. For assessment of cell invasion, MH7A cells were seeded at a density of 1 × 105 cells per chamber onto Matrigel-coated transwell plates in serum-free DMEM. The lower chamber contents and were the same as migration assay. After 24 h, cells on the top surface were removed, and invaded cells were imaged as described above.

### Statistical analysis

The data are presented in the form of Mean ± SD. Statistical methods used to compare differences were introduced in legend respectively.

## Results

### The preparation and characterization of HMSC-124a-EV

**Fig. 1:**
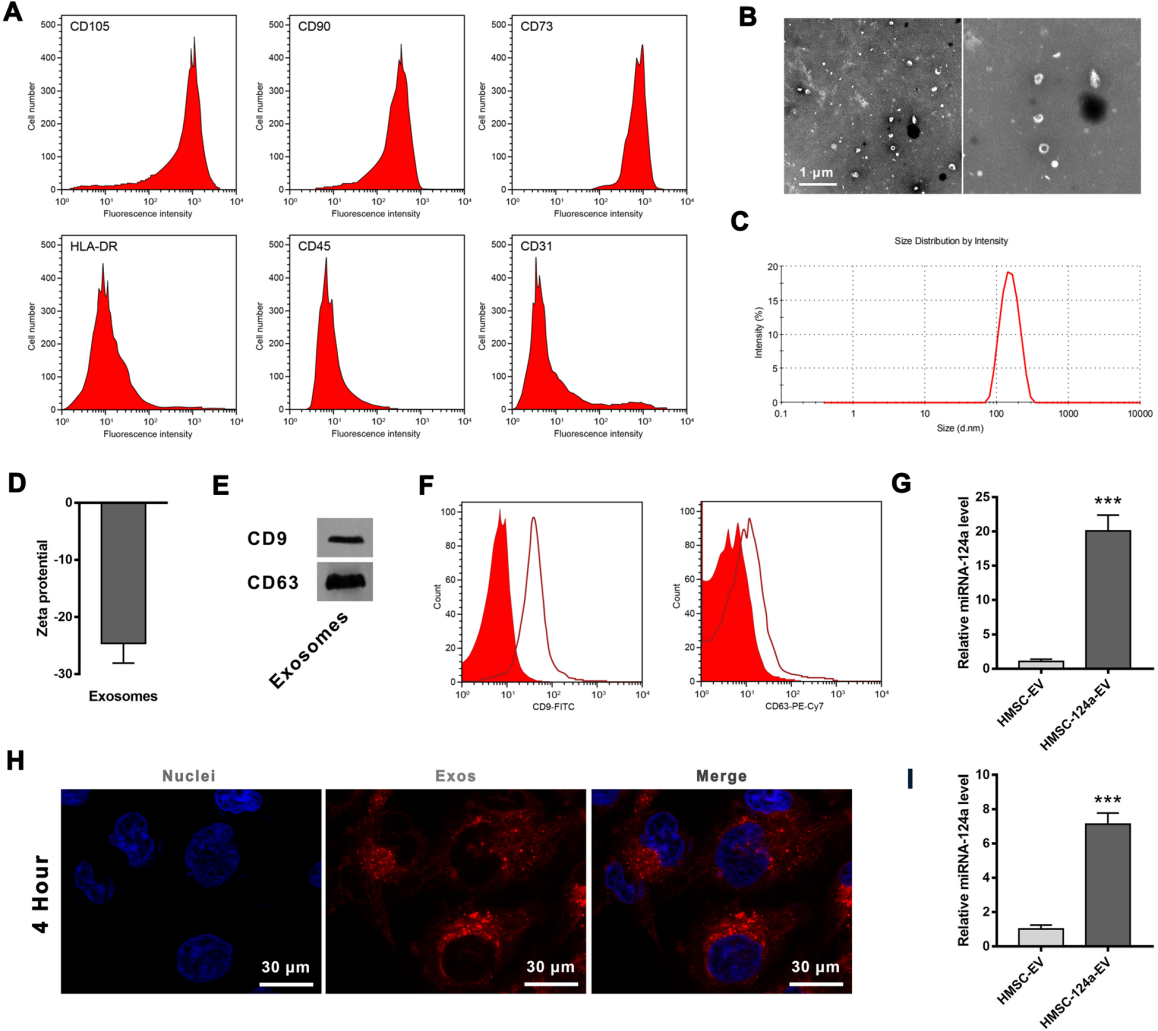
The preparation and characterization of HMSC-124a-EV. **a** The specificity biomarker of human MSC was detected by Flow cytometry; **b** TEM imaging of prepared exosomes; **c** The size distribution of exosome evaluated by Dynamic light scattering; **d** Zeta potential of the prepared HMSC-124a-EV; **e** The qualitative analysis of CD9 and CD63 within exosomes by western blot; **f** The detection of CD9 and CD63 by Flow cytometry; **g** Relative miRNA-124a level in HMSC-124a-EV measured by RT-PCR; **h** Fluorescent imaging of MH7A cells after the co-incubation with HMSC-124a-EV, blue represents nucleus and red represents the exosome phagocytosed by MH7A cell; **i** Relative miRNA-124a level in MH7A cells after the incubation with HMSC-124a-EV. *n* = 5, ***p < 0.001 as determined by Student’s t-test. All these data were representative of two independent experiments

Flow cytometry was employed to analyze the cell surface specific markers to identify the prepared MSCs (Fig. 1a). MSCs were able to express CD105, CD90, and CD73 but the HLA-DR, CD45, and CD31 were negative. As shown in Fig. 1b & c, TEM analysis and Dynamic light scattering (DLS) displayed vesicles around 100 nm diameter. The Zeta-protential of the exosome was 25.1 ± 5.1 (Fig. 1d). CD9 and CD63 that measured by Western Blot and flow cytometry was specific surface markers of exosomes. As shown in Fig. 1e & f, the level of CD9 and CD63 were readily detectable in the MSCs-derived exosome.

The level of miRNA-124a was detected by RT-PCR, and the result showed that HMSC-124a-EV was rich of miRNA-124a compare with HMSC-EV (human MSC-derived exosomes). After the characterization of HMSC-124a-EV, whether the exosome was able to delivery miRNA-124a into MH7A cell was confirmed by the imaging of fluorescence staining and RT-PCR. MH7A cells were chosen as a reliable in vitro model of the human rheumatoid fibroblast-like synoviocyte line for the well-characterization of this cell line and reliability. As shown in Fig. 1h & i, a large number of exosomes were found in MH7A cells, and high levels of miRNA-124a were detected in MH7A cells by the co-incubation of HMSC-124a-EV compared with the control group. Taken together, these results indicated the successful transportation of miRNA-124a into MH7A by HMSC-124a-EV.

### The proliferation inhibition and G0/G1 cell cycle arrest of MH7A after co-incubation with HMSC-124a-EV

**Fig. 2:**
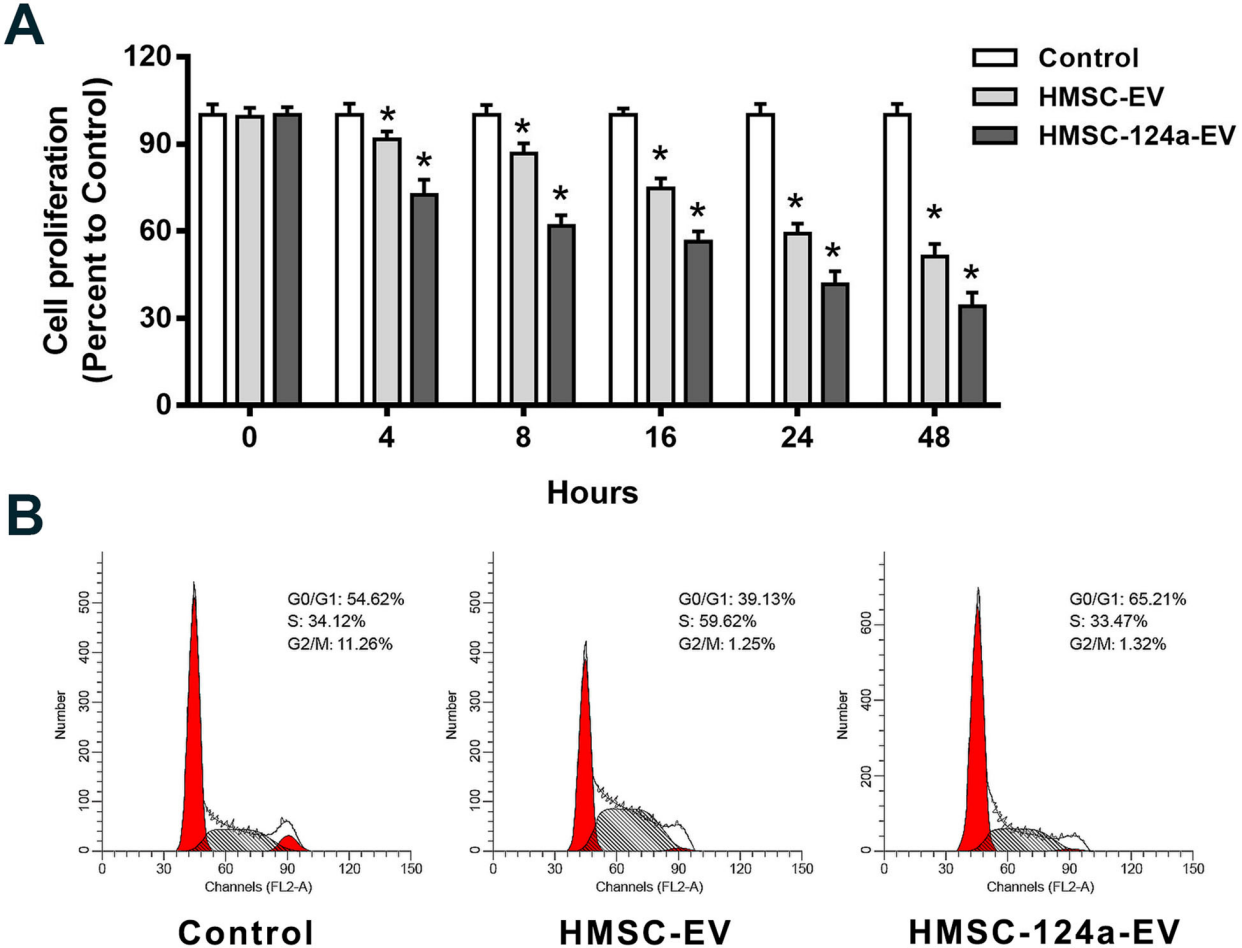
The inhibition of cell proliferation and cell cycle by HMSC-124a-EV. **a** MTT assay of the cell proliferation of MH7A at different time points after the co-incubation with HMSC-EV and HMSC-124a-EV, respectively; **b** Cell cycle detection of MH7A cells by above treatment. *n* = 5, * represent the comparison with the control group at the corresponding time points. *p* < 0.05 as determined by one-way ANOVA with the post-hoc Tukey’s test. All these data were representative of two independent experiments

Cell proliferation and cell cycle progression were measured by MTT and flow cytometry assay as shown in Fig. 2a & b. We found that HMSC-124a-EV significantly inhibits the proliferation of MH7A cells compared with HMSC-EV. The cell cycle detection by flow cytometry was aimed to reflect the percentage of cells in the different cell cycle phases such as G0/G1, S, and G2/M. As shown in Fig. 2b, the cells by HMSC-EV and HMSC-124a-EV treatment were blocked in the S phase and G0/G1 phase, respectively. There was no significant change in the percentage of G2/M-phase cells by HMSC-EV and HMSC-124a-EV treatment. All the above comparison was between the control group. Taken together, these results suggested that HMSC-124a-EV was able to evoke cell-cycle arrest at the G0/G1 phase and inhibit cell proliferation. It should be noted that HMSC-EV arrest the cells in S phase which may be the growth inhibition effect of exosomes form HMSCs and need to be further investigation.

### Scratch wound closure and cell migration of MH7A was reduced by HMSC-124a-EV

**Fig. 3:**
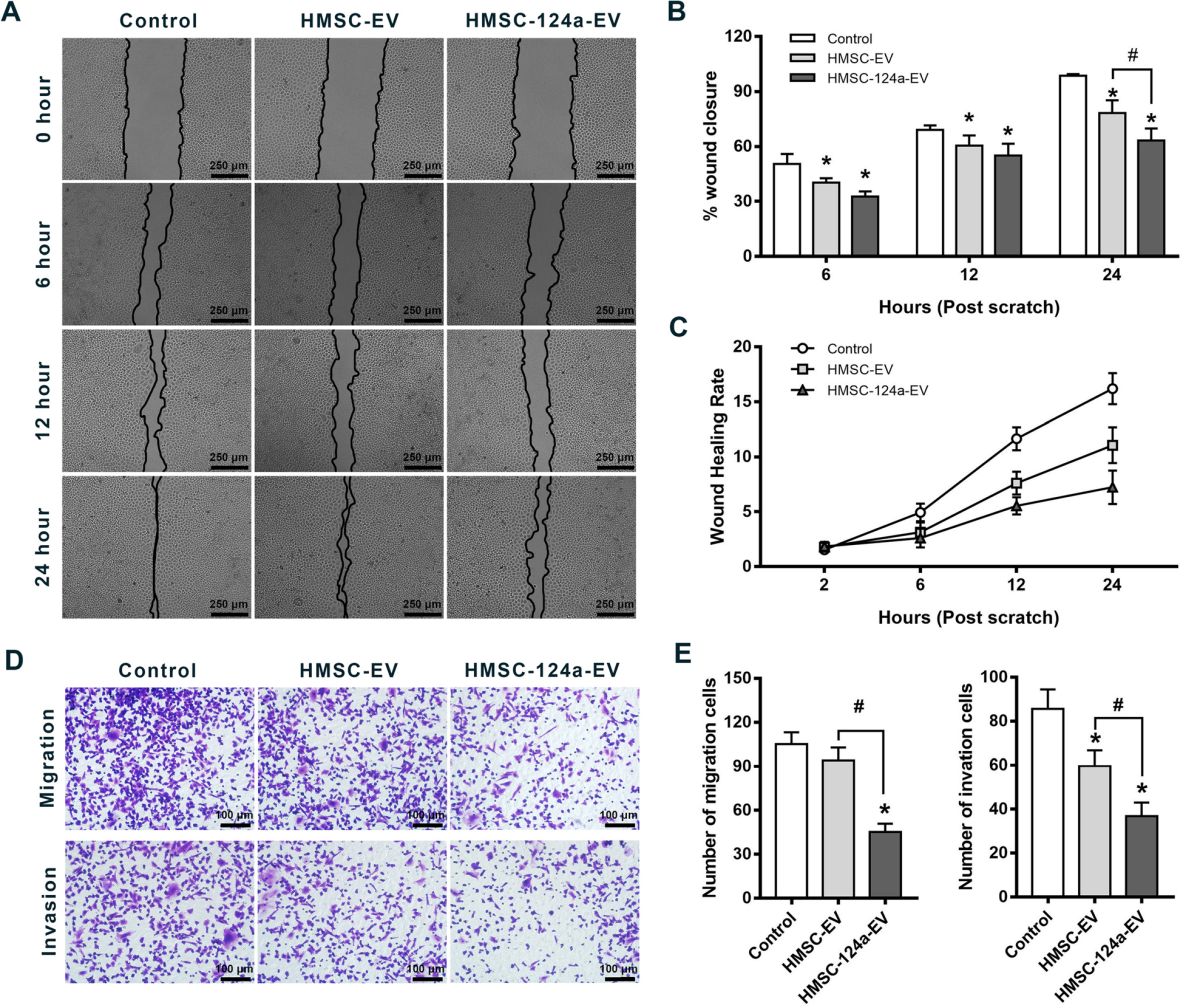
The attenuation of Scratch Wound Closure and Cell Migration by HMSC-124a-EV. **a** Representative images from Scratch Wound Closure assay of MH7A cells cultures treated with HMSC-EV and HMSC-124a-EV respectively; **b**&**c** Quantitative analysis of data in **a** about the percentage of wound closure and wound healing rates. *n* = 5, * represent the comparation with control group. *p < 0.05 and #*p* < 0.05 as determined by one-way ANOVA with the post-hoc Tukey’s test. **d** Migration and invasion assays in MH7A cells. Original magnification 100×; **e** The quantitative analysis of data in **d** about the numbers of migration or invasion cells. *n* = 5, * represent the comparation with control group. *p < 0.05 and #*p* < 0.05 as determined by one-way ANOVA with the post-hoc Tukey’s test. All these data were representative of two independent experiments

Scratch wound assay was used to evaluate the migration ability after co-incubation with HMSC-124a-EV in vitro. MH7A cells were successfully plated to confluence and then streaked to remove the same amounts of cells. The cells were subsequently treated with HMSC-EV and HMSC-124a-EV, respectively. Cell growth and migration rates were calculated by measuring cell coverage into the denuded space through imaging analysis. As shown in Fig. 3a, b & c, HMSC-124a-EV treatment induced an obvious inhibition of wound closure percentage and wound healing rate at 24 h compared with HMSC-EV and control group. It is worth noting that HMSC-EV treated group also has the ability to inhibit the Scratch Wound Closure, which was enhanced by the addition of miRNA-124a.

**Fig. 4:**
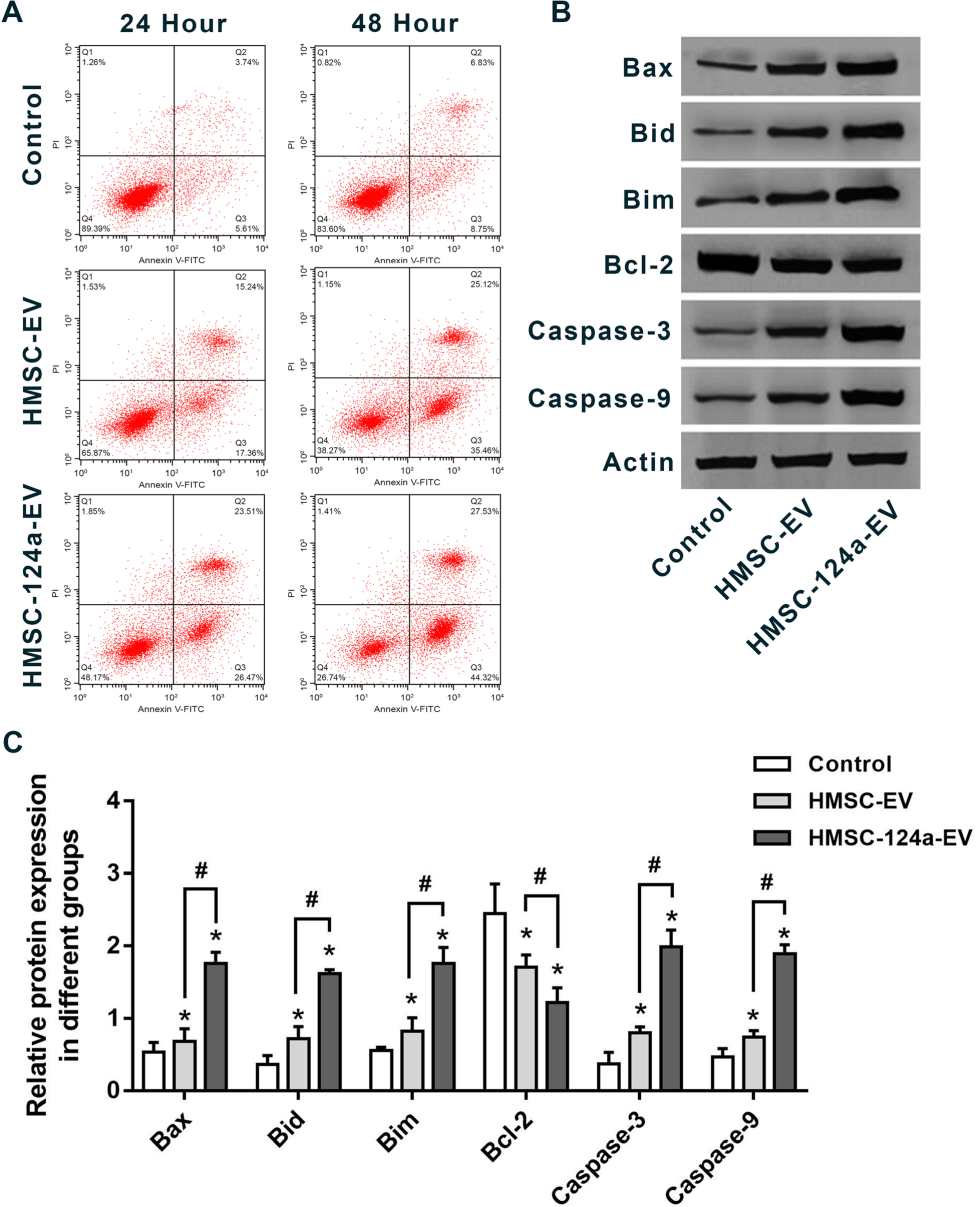
The induction of MH7A apoptosis by HMSC-124a-EV. **a** Flow cytometry analysis of cell apoptosis the co-stain with PI and Annexin-V; **b** The detection of apoptosis-related protein by western blot; **c** The quantitative analysis of data in **b**. *n* = 4. * represent the comparation with control group. *p < 0.05 and #*p* < 0.05 as determined by one-way ANOVA with the post-hoc Tukey’s test. All these data were representative of two independent experiments

In order to further evaluated the effect of HMSC-124a-EV on the migration ability of MH7A, we performed migration and invasion assays comparing cells after co-incubating with HMSC-124a-EV and HMSC-EV, respectively. As shown in Fig. 3c & d, HMSC-124a-EV treatment group significantly inhibited the migration and invasion of MH7A compared with the control and HMSC-EV group (Fig. 4a). A weaker scratch wound closure ability was detected by HMSC-EV co-incubation in the above result. However, there was no obvious change in migration and invasion ability of MH7A after the pretreatment of HMSC-EV. Taken together, these results suggested that miRNA-124a delivered by HMSC-EV was able to inhibit the scratch wound closure and migration of MH7A cell lines.

### The promotion of apoptosis in MH7A after co-incubation with HMSC-124a-EV

In this part, we investigated whether HMSC-124a-EV was able to induce apoptosis in MH7A cells. Flow cytometry and western blot were employed to study the apoptosis staining and apoptosis-related protein respectively. As shown in Fig. 4a, after 24 h and 48 h co-incubation with HMSC-124a-EV and HMSC-EV, respectively, the apoptosis was measured by the co-staining with PI and FITC-annexin V. An obvious promotion of apoptosis was found by the co-incubation with HMSC-124a-EV and HMSC-EV in MH7A at 24 h and 48 h, and the addition of miRNA-124a was aggravated the degree of apoptosis. Since the apoptotic cells increased with the co-incubation time,

we next detected the apoptosis-related protein including Bax, Bid, Bim, Bcl-2, caspase-3 and caspase-9 after 48 h co-incubation with HMSC-124a-EV and HMSC-EV. As shown in Fig. 4b & c, the concentration of Bax, Bid, Bim, caspase-3 and caspase-9 was obviously increased by the stimulation with HMSC-124a-EV compared with HMSC-EV and control group. Moreover, the co-incubation with HMSC-EV also increased the levels of this apoptosis-related protein, but weaker than HMSC-124a-EV group. Bcl-2, an anti-apoptosis protein, was down-regulated by the HMSC-124a-EV and HMSC-EV. The level of Bcl-2 in HMSC-EV was lower than that in the HMSC-124a-EV group, which was consistent well with the above results. Taken together, these results suggested that miRNA-124a was able to promote the apoptosis induction base on the delivery by HMSC-EV.

## Discussion

MSCs exert immunomodulatory effects on a variety of cells involved in both innate and adaptive immune responses [17]. Autologous MSCs was able to significantly suppress the proliferation of activated lymphocytes [23]. It has been reported that synovial MSCs extracted from healthy subjects can suppress the proliferation of T cells in vitro, possibly because it can suppress the proliferation of CD4^+^ cells and the expression of cytokines such as IL-2 and TNF-alpha [24]. Tse WT et al. reported the inhibition effect of allogeneic T cell proliferation by human MSCs in a RA mouse model [25]. The combination of allogeneic MSCs with conventional drugs can significantly ameliorate symptoms and improve serological in RA patients [26]. However, allogeneic MSCs have some problem in the treatment of RA. The survival and biological activity of mesenchymal stem cells are not easily controlled after entering the body, and there is a potential carcinogenic risk due to the inhibition of anti-tumor immune responses by MSCs therapy [19].

In the current work, exosomes derived from MSC (MSC-Exos) were used to partial replace MSC to avoid the adverse reaction. MSCs-Exos can transport a variety of biologically active proteins and RNA (mRNA, miRNA, and siRNA, etc.) to target cells to exert their biological functions [20]. In vitro experiments have shown that MSCs-Exos can exert immunosuppressive effects by inducing Treg cells migrated to inflammatory arthritis and reducing the proliferation of T and B lymphocytes [27]. In vivo experiments in animals have found that MSCs-Exos was able to alleviate the symptoms of osteoarthritis in osteoarthritic animals [28]. MSCs-Exos carrying miRNA-150-5p significantly down-regulated the concentration of MMP14 and VEGF (vascular endothelial growth factor) which reduced the severity of arthritis [16]. The design philosophy of the above research is the same as ours. In the current work, we generated exosomes form human MSC that overexpress miRNA-124a for the purpose of inhibiting the migration of FLS and inducing apoptosis. The ability of exosomes to transport small molecules, such as miRNA, between cells makes them effective therapeutic delivery vehicles and promising tools in the treatment of many diseases ranging from cancer to virus-induced and parasitic diseases [29, 30]. In addition, a large number of studies have been able to successfully prepare exosomes that overexpress miRNA and deliver them to target organs, making them valuable therapeutic tools [30]. The exosome used in our study, HMSC-124a-EV, combines the ability of exosomes to treat RA and the advantages of delivering miRNA. Through a series of in vitro experiments, our results indicate that the addition of miRNA-124a in exosomes was able to inhibit the migration of MH7A cells and promote apoptosis. Exosomes that do not carry miRNA also have similar functions, but the effect is not as obvious as HMSC-124a-EV treatment.

Early studies have shown that miRNA-124 is a mammalian nervous system-specific miRNA with three subtypes. miRNA-124a is one of them, which vitals in nervous system development, tumor metastases, and injury repair [31, 32]. However, recent study suggested that miRNA-124a are also existed in synoviocytes [33]. Seiji Kawano et al. reported that miR-124a was a key regulator of synoviocyte proliferation and MCP-1 secretion [15]. And the work of YanWang et al. indicated that the upregulation of miRNA-124a expression was able to inhibit TNF-α-stimulated cell proliferation in rheumatoid arthritis synovial fibroblasts [34]. Also, Liedao Yu et al. reported that miRNA-124a inhibits cell proliferation in Hepatic carcinoma by regulating IL-11 [13]. These researches consistent well with our results that HMSC-124a-EV obviously inhibited the proliferation of MH7A cell lines.

The migration of activated FLS is one of the decisive factors in the development of RA. The previous study suggested that down-regulated miR-124a in glioblastoma is related to the migration and invasion of the tumor [14], and similar results were obtained that miRNA-124a inhibits cell migration in liver cancer [13]. In our study, HMSC-124a-EV significant inhibited the migration of MH7A cell lines in the scratch wound closure and transwell assay, which in agreement with others’ research.

HMSC-124a-EV increased the expression of apoptosis-related protein such as Bax, Bid, Bim, caspase-3 and caspase-9, compared with the HMSC-EV group, which suggested that miRNA-124a was able to promote apoptosis. The previous study reported that miR-124a increased the expression of tissue inhibitor of Caspase-3 and matrix metalloproteinase-2 (TIMP-2, 13], which consistent well with our work. However, Nakanishi et al. found that the downregulation of miRNA-124a may be associated with neuronal cell apoptosis [35], and Liu NK et al. considered that apoptotic genes such as calpain 1, calpain 2, caspase-3 and apoptosis-inducing factor were potential targets of miR-124a, which were downregulated after spinal cord injury [36]. These results were different from our works, and the potential reason for this phenomenon may be the difference between nerve cells and synovial cells.

## Conclusions

We generated the exosomes from human MSCs that overexpress miRNA-124a, and found that co-incubation with HMSC-124a-EV was able to inhibit cell proliferation, migration, and promote the apoptosis in fibroblast-like synoviocyte cell line. Our data presented that exosomes derived from MSCs were a perfect vector for the delivering of therapeutic miRNA, which was expected to provide new medicine and strategy for the treatment of RA.

## Data Availability

The datasets generated and/or analyzed during the current study are available from the corresponding author by reasonable request.

## Abbreviations

DMARDs: disease-modifying anti-rheumatic drugs
FLS: Fibroblast-like synoviocytes
HMSC-124a-EV: Human mesenchymal stem cells derived exosomes that overexpression miRNA-124a
HMSC-EV: Human mesenchymal stem cells derived exosomes
MCP1: Monocyte Chemoattractant Protein 1
MSCs: Mesenchymal stem cells
RA: Rheumatoid arthritis
UC-MSCs: umbilical cord mesenchymal stem cells
